# Ayurveda for managing noncommunicable diseases in organisation for economic co‐operation and development: A qualitative systematic review protocol on experiences, perceptions, and perspectives of ayurvedic practitioners and patients

**DOI:** 10.1002/hsr2.1530

**Published:** 2023-09-14

**Authors:** Patricia Egwumba, Laura Nellums, Manpreet Bains, Kaushik Chattopadhyay

**Affiliations:** ^1^ Lifespan and Population Health Academic Unit, School of Medicine University of Nottingham Nottingham UK; ^2^ The Nottingham Centre for Evidence‐Based Healthcare: a JBI Centre of Excellence Nottingham UK

**Keywords:** ayurveda, management, noncommunicable diseases, OECD, qualitative systematic review

## Abstract

**Background and Aims:**

Ayurveda is a traditional medicine that originated in the Indian subcontinent, and its use remains widespread in the Indian subcontinent, especially for managing noncommunicable diseases (NCDs). It is also becoming increasingly popular in the Organization for Economic Co‐operation and Development (OECD) countries as complementary and alternative medicine. Qualitative research studies have been conducted in various OECD countries to explore the experiences, perceptions, and perspectives of Ayurvedic practitioners and patients with NCDs regarding the usage of Ayurveda for managing these conditions. However, to date, no systematic review on this topic has been published. Therefore, this systematic review aims to synthesize the experiences, perceptions, and perspectives of Ayurvedic practitioners and patients with NCDs on the usage of Ayurveda for managing these conditions in OECD countries.

**Methods:**

The systematic review will be conducted in accordance with the joanna briggs institute systematic review guideline on qualitative evidence. We will include qualitative research studies conducted among Ayurvedic practitioners or adult patients with NCDs in any OECD member country to explore experiences, perceptions, or perspectives regarding the usage of Ayurveda for managing NCDs. MEDLINE (Ovid), Embase (Ovid), CINAHL (EBSCOhost), PsycINFO (Ovid), AMED, and Web of Science will be searched to identify published studies. EthOS and ProQuest Dissertations and Theses will be searched to identify unpublished studies. No date or language restrictions will be applied. Initially, a narrative synthesis will be conducted. Where possible, study findings will be pooled using the meta‐aggregation approach.

## Key points

- Ayurveda is one of the oldest medical systems in the world.
- The spread of Ayurveda is partially a result of the migration of individuals from the Indian subcontinent to other countries.
- Ancient texts on Ayurveda have vividly described the management of several noncommunicable disease (NCD).
- Systematic reviews of clinical trials have demonstrated the effectiveness and safety of Ayurveda for managing NCDs.

## 1 INTRODUCTION

### 1.1 Burden of noncommunicable diseases (NCDs) in organization for economic co‐operation and development

The Organization for Economic Co‐operation and Development (OECD) is an intergovernmental organization with 38 member countries.^1^ Most OECD nations are high‐income countries with a high Human Development Index (HDI).^1^ One of the major public health interests of the OECD is to identify, plan, and implement effective interventions to manage NCDs.^2^ NCDs are conditions that have long‐term health and socioeconomic consequences and often necessitate a need for long‐term management.^3^, ^4^ Due to changes in the population structure, environment, and lifestyle and behavior, the prevalence of NCDs has rapidly increased in many OECD countries.^5^ NCDs are the leading cause of morbidity and poor quality of life in several OECD countries.^5^ In 2019, compared with the global average of 80%, NCDs accounted for 88% of all years lived with disability (YLDs) in OECD countries.^6^ In OECD countries, 85% of all disability‐adjusted life years (DALYs) were attributed to NCDs, compared with the global average of 64%.^6^ Similarly, NCDs are the leading cause of death in many OECD countries, and in 2019, 89% of total deaths in OECD countries were attributed to NCDs, which was higher than the global average of 74%.^6^

In OECD countries, NCDs cause increased demand for health services, and the high treatment costs lead to increased health expenditures.^5^, ^7^, ^8^ Furthermore, NCDs in OECD countries cause significant productivity loss due to absenteeism (unable to work due to illness), presenteeism (working less effectively), and early retirement.^5^, ^7^, ^8^ A comprehensive evaluation in OECD nations reported that compared with individuals who did not report having an NCD in a particular year, those having at least one NCD were less likely to be employed in the following year.^9^ Moreover, those with at least two NCDs had an even lower likelihood of being employed in the following year and were more likely to retire early.^9^

### 1.2 Ayurveda for NCD management in organization for economic co‐operation and development

Ayurveda, which means “knowledge of life,” is a traditional medicine that originated in the Indian subcontinent more than 5000 years ago and is one of the oldest medical systems in the world.^10^, ^11^ It aims to preserve health by following a healthy lifestyle and to manage diseases if they occur.^11^ Ayurveda is considered a complex intervention, which uses Ayurvedic detoxifying and purifying therapies (e.g., Panchakarma) and Ayurvedic medicines (containing plant‐, animal‐, or mineral‐origin ingredients–single or in combination).^12^ The mechanism of action depends on the specific therapy or medicine and the health condition in which it is used.^11^ Ayurveda's use remains widespread in the Indian subcontinent because it aligns with their health beliefs and culture, and as a result, its acceptability, satisfaction, and perceived relief are generally high, particularly among the poor, older, rural, and tribal populations.^12^ Ancient texts on Ayurveda have vividly described the management of several NCDs.^13^, ^14^, ^15^ and these texts are followed by Ayurvedic practitioners to manage these conditions in real practice.^16^, ^17^ In the modern era, several systematic reviews of clinical trials have been conducted on the effectiveness and safety of Ayurveda for managing NCDs.^18^, ^19^, ^20^, ^21^, ^22^, ^23^ For example, a systematic review reported the effectiveness and safety of several Ayurvedic medicines for managing type 2 diabetes mellitus.^18^ Another systematic review reported the effectiveness and safety of Ayurvedic herbal preparations for managing hypercholesterolemia.^19^ Ayurveda is frequently perceived as safe by Ayurvedic practitioners and people.^24^ However, cases of heavy metal (such as mercury and lead) poisoning have been reported after using some Ayurvedic medicines that contain heavy metals.^25^ Many Ayurvedic practitioners assert that if the precise heavy metal processing methods, mentioned in Ayurvedic classical texts, are not followed, heavy metal poisoning will most likely occur.^12^

The World Health Organization (WHO) defines complementary and alternative medicine (CAM) as a broad set of healthcare practices that are not part of a country's own tradition or conventional medicine and are not fully integrated into the dominant healthcare system.^26^ Ayurveda is considered CAM in many OECD countries.^26^, ^27^ Moreover, Ayurveda is now officially recognized in five OECD countries: Colombia, Switzerland, Hungary, Latvia, and Slovenia.^28^ Ayurveda is becoming increasingly popular in many OECD countries, such as the United Kingdom (UK), the United States of America (USA), and Canada, and it is one of the fastest‐growing CAMs in Germany and Austria.^29^, ^30^, ^31^, ^32^, ^33^, ^34^ The spread of Ayurveda in these countries is partially a result of the migration of individuals from the Indian subcontinent to these countries and the growing interest of local people in Ayurveda.^32^, ^34^ For instance, it has been reported that South Asian migrants in the USA and Canada have used Ayurvedic medicines at some point in the past to treat various medical conditions, including NCDs.^34^, ^35^ Some other reasons for choosing Ayurveda to manage NCDs in OECD nations are patients' dissatisfaction with the NCD management approach of Western medical practitioners (which includes perceived side effects of Western medicines) and pleasant experiences during Ayurvedic consultations compared with Western medical consultations.^32^, ^33^, ^34^, ^36^

### 1.3 Rationale for the systematic review

Qualitative research studies have been conducted in various OECD countries to explore the experiences, perceptions, and perspectives of Ayurvedic practitioners and patients with NCDs on the usage of Ayurveda for managing these conditions.^32^, ^33^, ^34^, ^36^ However, to date, no systematic review on this topic has been published, and this systematic review aims to synthesize these experiences, perceptions, and perspectives.

### 1.4 Review question

What experiences, perceptions, and perspectives do Ayurvedic practitioners and patients with NCDs have on the usage of Ayurveda for managing these conditions in OECD countries?

## 2 METHODS

The systematic review will be conducted according to the joanna briggs institute (JBI) systematic review guideline on qualitative evidence^37^ and reported according to the enhancing transparency in reporting the synthesis of qualitative research (ENTREQ) guideline.^38^ The systematic review protocol has been registered with PROSPERO (CRD42023397952).

### 2.1 Inclusion criteria

#### 2.1.1 Participants

The systematic review will include studies conducted among Ayurvedic practitioners or adult ( ≥ 18 years old) patients with NCDs.

#### 2.1.2 Phenomena of interest

This review will include studies that explored experiences, perceptions, or perspectives regarding the usage of Ayurveda for managing NCDs.

#### 2.1.3 Context

This review will include studies conducted in any OECD member country, namely, Australia, Austria, Belgium, Canada, Chile, Colombia, Costa Rica, Czech Republic, Denmark, Estonia, Finland, France, Germany, Greece, Hungary, Iceland, Ireland, Israel, Italy, Japan, Korea, Latvia, Lithuania, Luxembourg, Mexico, Netherlands, New Zealand, Norway, Poland, Portugal, Slovakia, Slovenia, Spain, Sweden, Switzerland, Turkey, UK, and USA.^1^ Any study setting will be eligible, such as community, primary care, secondary care, or tertiary care.

#### 2.1.4 Type of studies

This review will include qualitative research studies that used data collection methods like semistructured interviews, focus group discussions, observational studies, ethnographic studies, documents, case note analyses, or diaries.

### 2.2 Databases and search strategies

The following databases will be searched for published studies: MEDLINE (Ovid; 1946‐present), Embase (Ovid; 1974‐present), CINAHL (EBSCOhost; 1961–present), PsycINFO (1806–present), AMED (1985–present), and Web of Science (1900–present). The search for unpublished studies will include EthOS and ProQuest Dissertations and Theses. The search strategies are reported in Table 1. The search strategies were developed in consultation with a research librarian at the University of Nottingham (UK). Ayurveda and qualitative study design components are based on the search strategies used in previous systematic reviews.^18^, ^39^, ^40^ The reference list of the included studies and previous relevant systematic reviews will be screened for additional studies. No language restrictions will be applied, and translations will be sought where necessary. Similarly, no date restrictions will be applied.

**Table 1:** Search strategies.

*MEDLINE (Ovid)* 1 exp Australia/ 2 exp Austria/ 3 exp Belgium/ 4 exp Canada/ 5 exp Chile/ 6 exp Colombia/ 7 exp Costa Rica/ 8 exp Czech Republic/ 9 exp Denmark/ 10 exp Estonia/ 11 exp Finland/ 12 exp France/ 13 exp Germany/ 14 exp Greece/ 15 exp Hungary/ 16 exp Iceland/ 17 exp Ireland/ 18 exp Israel/ 19 exp Italy/ 20 exp Japan/ 21 exp Latvia/ 22 exp Lithuania/ 23 exp Luxembourg/ 24 exp Netherlands/ 25 exp New Zealand/ 26 exp Norway/ 27 exp Poland/ 28 exp Slovakia/ 29 exp Slovenia/ 30 exp Spain/ 31 exp Sweden/ 32 exp Switzerland/ 33 exp Turkey/ 34 exp United Kingdom/ 35 exp England/ 36 exp Northern Ireland/ 37 exp Scotland/ 38 exp Wales/ 39 exp United States/ 40 exp “Democratic People's Republic of Korea”/ 41 exp “Republic of Korea”/ 42 exp Korea/ 43 exp Mexico/ 44 exp Portugal/ 45 exp “Organisation for Economic Co‐Operation and Development”/ 46 (Australia or Austria or Belgium or Canada or Chile or Colombia or Costa Rica or Czech Republic or Denmark or Estonia or Finland or France or Germany or Greece or Hungary or Iceland or Ireland or Israel or Italy or Japan or Latvia or Lithuania or Luxembourg or Netherlands or Holland or New Zealand or Norway or Poland or Slovakia or Portugal or Slovenia or Spain or Sweden or Switzerland or Turkey or United Kingdom or UK or Britain or Great Britain or England or Northern Ireland or Scotland or Wales or United States or USA or United States of America or Republic of Korea or Democratic People's Republic of Korea or Korea or Mexico or OECD). mp. 47 1 or 2 or 3 or 4 or 5 or 6 or 7 or 8 or 9 or 10 or 11 or 12 or 13 or 14 or 15 or 16 or 17 or 18 or 19 or 20 or 21 or 22 or 23 or 24 or 25 or 26 or 27 or 28 or 29 or 30 or 31 or 32 or 33 or 34 or 35 or 36 or 37 or 38 or 39 or 40 or 41 or 42 or 43 or 44 or 45 or 46 48 exp Medicine, Ayurvedic/ 49 Ayurved*. tw, ot. 50 *Medicine, Traditional/ 51 exp Complementary medicine/tu [Therapeutic use] 52 ((plant* or herb* or medicin* or drug*or therap*or intervention*or extract* or formulation*or preparation* or supplement*) adj6 (Ayurved* or Hindu or Indian)). tw, ot. 53 exp Plants, Medicinal/tu [Therapeutic use] 54 exp Plant Extracts/tu [Therapeutic use] 55 exp Plants/tu [Therapeutic use] 56 ((plant* or herb*) adj6 (medicin* or drug* or therap* or intervention* or extract* or formulation* or preparation* or supplement*)). tw, ot. 57 exp Ethnobotany/ 58 exp Ethnopharmacology/ 59 (ethnobotan* or ethno botan* or ethnopharmacolog* or ethno pharmacolog*). tw, ot. 60 *Phytotherapy/ 61 (phytotherap* or phyto therap*). tw, ot. 62 (Complementary and alternative medicine). mp. 63 CAM. mp. 64 48 or 49 or 50 or 51 or 52 or 53 or 54 or 55 or 56 or 57 or 58 or 59 or 60 or 61 or 62 or 63 65 exp Qualitative Research/ 66 exp Interview/ 67 exp Focus Groups/ 68 exp Observation/ 69 exp Case Reports/ 70 exp Grounded Theory/ 71 (qualitative* or interview* or focus group* or observations*).mp. 72 (Grounded Theory or Phenomenology* or Ethnograph* or Action research or Narrative research).mp. 73 (mixed method studies or qualitative systematic reviews).mp. 74 Documents.mp. 75 65 or 66 or 67 or 68 or 69 or 70 or 71 or 72 or 73 or 74 76 47 and 64 and 75
*Embase (Ovid)* 1 exp Australia/ 2 exp Austria/ 3 exp Belgium/ 4 exp Canada/ 5 exp Chile/ 6 exp Colombia/ 7 exp Costa Rica/ 8 exp Czech Republic/ 9 exp Denmark/ 10 exp Estonia/ 11 exp Finland/ 12 exp France/ 13 exp Germany/ 14 exp Greece/ 15 exp Hungary/ 16 exp Iceland/ 17 exp Ireland/ 18 exp Israel/ 19 exp Italy/ 20 exp Japan/ 21 exp Latvia/ 22 exp Lithuania/ 23 exp Luxembourg/ 24 exp Netherlands/ 25 exp New Zealand/ 26 exp Norway/ 27 exp Poland/ 28 exp Slovakia/ 29 exp Slovenia/ 30 exp Spain/ 31 exp Sweden/ 32 exp Switzerland/ 33 exp United Kingdom/ 34 exp England/ 35 exp Northern Ireland/ 36 exp Scotland/ 37 exp Wales/ 38 exp United States/ 39 exp “Democratic People's Republic of Korea”/ 40 exp “Republic of Korea”/ 41 exp Korea/ 42 exp Mexico/ 43 exp Portugal/ 44 exp “Turkey (republic)”/ 45 exp “Organisation for Economic Co‐operation and Development”/ 46 (Australia or Austria or Belgium or Canada or Chile or Colombia or Costa Rica or Czech Republic or Denmark or Estonia or Finland or France or Germany or Greece or Hungary or Iceland or Ireland or Israel or Italy or Japan or Latvia or Lithuania or Luxembourg or Netherlands or Holland or New Zealand or Norway or Poland or Slovakia or Portugal or Slovenia or Spain or Sweden or Switzerland or Turkey or United Kingdom or UK or Britain or Great Britain or England or Northern Ireland or Scotland or Wales or United States or USA or United States of America or Republic of Korea or Democratic People's Republic of Korea or Korea or Mexico or OECD).mp. 47 1 or 2 or 3 or 4 or 5 or 6 or 7 or 8 or 9 or 10 or 11 or 12 or 13 or 14 or 15 or 16 or 17 or 18 or 19 or 20 or 21 or 22 or 23 or 24 or 25 or 26 or 27 or 28 or 29 or 30 or 31 or 32 or 33 or 34 or 35 or 36 or 37 or 38 or 39 or 40 or 41 or 42 or 43 or 44 or 45 or 46 48 exp Ayurveda/ 49 Ayurved*.tw,ot. 50 *Medicine, Traditional/ 51 exp alternative medicine/ 52 ((plant* or herb* or medicin* or drug*or therap*or intervention*or extract* or formulation*or preparation* or supplement*) adj6 (Ayurved* or Hindu or Indian)).tw,ot. 53 exp medicinal plant/ 54 exp plant extract/ 55 ((plant* or herb*) adj6 (medicin* or drug* or therap* or intervention* or extract* or formulation* or preparation* or supplement*)).tw,ot. 56 exp ethnobotany/ 57 exp ethnopharmacology/ 58 (ethnobotan* or ethno botan* or ethnopharmacolog* or ethno pharmacolog*).tw,ot. 59 *Phytotherapy/ 60 (phytotherap* or phyto therap*).tw,ot. 61 (Complementary and alternative medicine). mp. 62 CAM.mp. 63 48 or 49 or 50 or 51 or 52 or 53 or 54 or 55 or 56 or 57 or 58 or 59 or 60 or 61 or 62 64 exp qualitative research/ 65 exp interview/ 66 exp observation/ 67 exp case report/ 68 exp grounded theory/ 69 (qualitative* or interview* or focus group* or observations*).mp. 70 (Grounded Theory or Phenomenology* or Ethnograph* or Action research or Narrative research).mp. 71 (mixed method studies or qualitative systematic reviews).mp. 72 Documents.mp. 73 64 or 65 or 66 or 67 or 68 or 69 or 70 or 71 or 72 74 47 and 63 and 73
*CINAHL (EBSCOhost)* 1 TX (Australia or Austria or Belgium or Canada or Chile or Colombia or Costa Rica or Czech Republic or Denmark or Estonia or Finland or France or Germany or Greece or Hungary or Iceland or Ireland or Israel or Italy or Japan or Latvia or Lithuania or Luxembourg or Netherlands or Holland or New Zealand or Norway or Poland or Slovakia or Portugal or Slovenia or Spain or Sweden or Switzerland or Turkey or United Kingdom or UK or Britain or Great Britain or England or Northern Ireland or Scotland or Wales or United States or USA or United States of America or Republic of Korea or Democratic People's Republic of Korea or Korea or Mexico or OECD)/S1 2 (MH “Medicine, Ayurvedic”)/S2 3 TX (medicine, Ayurvedic or Ayurved*)/S3 4 S2 or S3/S4 5 (MH “Qualitative Studies + ”)/S5 6 (MH “Interviews + ”)/S6 7 (MH “Focus Groups”)/S7 8 TX (qualitative or interview* or focus group*)/S8 9 TX (mixed method studies or qualitative systematic reviews)/S9 10 (MH “Grounded Theory”)/S10 11 (MH “Phenomenology”)/S11 12 (MH “Ethnographic Research”)/S12 13 (MH “Action Research”)/S13 14 TX (Grounded Theory or Phenomenology* or Ethnographic research or Action research)/S14 15 S5 or S6 or S7 or S8 or S9 or S10 or S11 or S12 or S13 or S14/S15 16 S1 and S4 and S15
*PsycINFO (Ovid)* 1 “Organisation for Economic Co‐Operation and Development”.mp. 2 (Australia or Austria or Belgium or Canada or Chile or Colombia or Costa Rica or Czech Republic or Denmark or Estonia or Finland or France or Germany or Greece or Hungary or Iceland or Ireland or Israel or Italy or Japan or Latvia or Lithuania or Luxembourg or Netherlands or Holland or New Zealand or Norway or Poland or Slovakia or Portugal or Slovenia or Spain or Sweden or Switzerland or Turkey or United Kingdom or UK or Britain or Great Britain or England or Northern Ireland or Scotland or Wales or United States or USA or United States of America or Republic of Korea or Democratic People's Republic of Korea or Korea or Mexico or OECD).mp. 3 1 or 2 4 exp “Medicinal Herbs and Plants”/ 5 exp Alternative Medicine/ 6 Ayurved* tw, ot. 7 ((plant* or herb*) adj6 (medicin* or drug* or therap* or intervention* or extract* or formulation* or preparation* or supplement*)) tw,ot. 8 (ethnobotan* or ethno botan* or ethnopharmacolog* or ethno pharmacolog*) tw,ot. 9 (phytotherap* or phyto therap*).tw ot. 10 (Complementary and alternative medicine). mp. 11 CAM.mp. 12 4 or 5 or 6 or 7 or 8 or 9 or 10 or 11 13 exp Qualitative Methods/ 14 exp Interviews/ 15 exp Focus Group/ 16 exp Participant Observation/ 17 exp Case Report/ 18 exp Grounded Theory/ 19 (qualitative* or interview* or focus group* or observations*).mp. 20 (Grounded Theory or Phenomenology* or Ethnograph* or Action research or Narrative research).mp. 21 (mixed method studies or qualitative systematic reviews).mp. 22 Documents.mp. 23 13 or 14 or 15 or 16 or 17 or 18 or 19 or 20 or 21 or 22 24 3 and 12 and 23
*Allied and Complementary Medicine Database (AMED) (Ovid)* 1 exp Australia/ 2 exp Canada/ 3 exp Denmark/ 4 exp Finland/ 5 exp France/ 6 exp Germany/ 7 exp Hungary/ 8 exp Iceland/ 9 exp Ireland/ 10 exp Israel/ 11 exp Italy/ 12 exp Japan/ 13 exp Netherlands/ 14 exp New Zealand/ 15 exp Poland/ 16 exp Spain/ 17 exp Sweden/ 18 exp Switzerland/ 19 exp England/ 20 exp Northern Ireland/ 21 exp Scotland/ 22 exp Wales/ 23 exp United States/ 24 exp Korea/ 25 exp Mexico/ 26 “Organisation for Economic Co‐Operation and Development”.mp. 27 (Australia or Austria or Belgium or Canada or Chile or Colombia or Costa Rica or Czech Republic or Denmark or Estonia or Finland or France or Germany or Greece or Hungary or Iceland or Ireland or Israel or Italy or Japan or Latvia or Lithuania or Luxembourg or Netherlands or Holland or New Zealand or Norway or Poland or Slovakia or Portugal or Slovenia or Spain or Sweden or Switzerland or Turkey or United Kingdom or UK or Britain or Great Britain or England or Northern Ireland or Scotland or Wales or United States or USA or United States of America or Republic of Korea or Democratic People's Republic of Korea or Korea or Mexico or OECD).mp. 28 1 or 2 or 3 or 4 or 5 or 6 or 7 or 8 or 9 or 10 or 11 or 12 or 13 or 14 or 15 or 16 or 17 or 18 or 19 or 20 or 21 or 22 or 23 or 24 or 25 or 26 or 27 29 exp Ayurvedic medicine/ 30 exp Plants medicinal/ 31 exp Plant extracts/ 32 exp Ethnopharmacology/ 33 (Complementary and alternative medicine).mp. 34 CAM.mp. 35 exp Traditional medicine/ 36 exp Complementary medicine/ 37 exp Complementary therapies/ 38 29 or 30 or 31 or 32 or 33 or 34 or 35 or 36 or 37 39 exp Interviews/ 40 exp Case report/ 41 (qualitative* or interview* or focus group* or observations*).mp. 42 (Grounded Theory or Phenomenology* or Ethnograph* or Action research or Narrative research).mp. 43 (mixed method studies or qualitative systematic reviews).mp. 44 Documents.mp. 45 39 or 40 or 41 or 42 or 43 or 44 46 4628 and 38 and 45
*Web of Science* #1 TI= (Australia or Austria or Belgium or Canada or Chile or Colombia or Costa Rica or Czech Republic or Denmark or Estonia or Finland or France or Germany or Greece or Hungary or Iceland or Ireland or Israel or Italy or Japan or Latvia or Lithuania or Luxembourg or Netherlands or Holland or New Zealand or Norway or Poland or Slovakia or Portugal or Slovenia or Spain or Sweden or Switzerland or Turkey or United Kingdom or UK or Britain or Great Britain or England or Northern Ireland or Scotland or Wales or United States or USA or United States of America or Republic of Korea or Democratic People's Republic of Korea or Korea or Mexico or OECD) #2 TI= (“Ayurvedic medicine” or Ayurved* or “traditional medicine” or “complementary medicine” or ((plant* or herb* or medicin* or drug* or therap* or intervention* or extract* or formulation* or preparation* or supplement*) adj6 (Ayurved* or Hindu or Indian)) or “medicinal plants” or “plant extracts” or plants or ((plant* or herb*) adj6 (medicin* or drug* or therap* or intervention* or extract* or formulation* or preparation* or supplement*)) or ethnobotany or ethnopharmacology or (ethnobotan* or ethno botan* or ethnopharmacolog* or ethno pharmacolog*) or phytotherapy or (phytotherap* or phyto therap*)) #3 ALL= (Case reports or (Qualitative*or interview*or focus group*or observations*) or (grounded theory or phenomenology* or ethnograph*) or (mixed method studies or qualitative systematic reviews) or documents) #1 and #2 and #3
*EthOS* Ayurveda [any word] or Ayurvedic [any word]
*ProQuest Dissertations and Theses* Ayurved* and summary((Australia or Austria or Belgium or Canada or Chile or Colombia or Costa Rica or Czech Republic or Denmark or Estonia or Finland or France or Germany or Greece or Hungary or Iceland or Ireland or Israel or Italy or Japan or Latvia or Lithuania or Luxembourg or Netherlands or Holland or New Zealand or Norway or Poland or Slovakia or Portugal or Slovenia or Spain or Sweden or Switzerland or Turkey or United Kingdom or UK or Britain or Great Britain or England or Northern Ireland or Scotland or Wales or United States or USA or United States of America or Republic of Korea or Democratic People's Republic of Korea or Korea or Mexico or OECD))

### 2.3 Study selection

Following the search, all citations identified will be collated and uploaded into EndNote X9 (Clarivate Analytics)^41^ a reference management software. Subsequently, the duplicate citations will be removed. Two reviewers (P. E. and K. C./L. N./M. B.) will independently screen titles and abstracts for eligibility using the systematic review inclusion criteria. Studies identified as potentially eligible or those without an abstract will have their full text retrieved, and their details will be imported into the JBI system for the unified management, assessment, and review of information (JBI SUMARI, JBI).^42^ Two reviewers (P. E. and K. C./L. N./M. B.) will independently assess the full text of studies against the inclusion criteria. Full‐text studies that do not meet the inclusion criteria will be excluded, and the reasons for exclusion will be reported. Any disagreements that arise between the two reviewers will be resolved through discussion. If a consensus is not reached, a third reviewer (K. C./L. N./M. B.) will be involved.

### 2.4 Assessment of methodological quality

Two reviewers (P. E. and K. C./L. N./M. B.) will independently assess the methodological quality of the included studies using the standardized critical appraisal tool incorporated within JBI SUMARI for qualitative research.^37^ This tool uses a series of criteria that can be scored as being met (yes), not met (no), unclear, or, where appropriate, not applicable (n/a) to that particular study. Any disagreements that arise between the reviewers will be resolved through discussion. If no consensus is reached, a third reviewer (K. C./L. N./M. B.) will be involved. Where possible, all included studies, regardless of the methodological quality assessment outcome, will undergo data extraction and synthesis.

### 2.5 Data extraction

Two reviewers (P. E. and K. C./L. N./M. B.) will independently extract data from the included studies using the standardized data extraction tool incorporated within JBI SUMARI.^37^ Any disagreements that arise between the reviewers will be resolved through discussion. If a consensus is not reached, a third reviewer (K. C./L. N./M. B.) will be involved. The following data will be extracted: author, year of publication, study period, participants (Ayurvedic practitioners or patients with NCDs), participant characteristics (such as sample size and their age and sex), OECD country, study setting (such as community, primary care, secondary care, or tertiary care), qualitative research methodology, data collection methods (such as semistructured interviews, focus group discussions, observational studies, ethnographic studies, documents, case note analyses, or diaries), data analyses technique, and study findings (i.e., experiences, perceptions, or perspectives on the usage of Ayurveda for managing NCDs). Each extracted finding will be supported by a verbatim data excerpt from the study's participants. Where this is not possible, the author's narrative will be extracted. These findings will be reviewed and assigned a level of credibility based on the JBI guideline:

1. Unequivocal: the finding is accompanied by an illustration that is beyond a reasonable doubt and is not open to challenge.
2. Credible: the finding is accompanied by an illustration that lacks a clear association with it and is open to challenge.
3. Not supported: when neither unequivocal nor credible can be applied and when the most notable findings are not supported by the data.^37^

### 2.6 Data synthesis

To uncover any similarities and differences between Ayurvedic practitioners and patients with NCDs in terms of their experiences, perceptions, and perspectives regarding the usage of Ayurveda for managing NCDs in OECD countries, their data will be synthesized separately. Initially, a narrative synthesis will be conducted. Where possible, study findings will be pooled using the meta‐aggregation approach.^37^, ^43^ This will involve the aggregation or synthesis of findings to generate a set of statements representing that aggregation, which will be accomplished by assembling the findings and categorizing them on the basis of their similarity in meaning. Next, these categories will be synthesized to generate a single comprehensive set of synthesized findings.

## AUTHOR CONTRIBUTIONS

**Patricia Egwumba**: Conceptualization; methodology; project administration; resources; visualization; writing—original draft; writing—review & editing. **Laura Nellums**: Methodology; supervision; writing—original draft; writing—review & editing. **Manpreet Bains**: Methodology; supervision; writing—original draft; writing—review & editing. **Kaushik Chattopadhyay**: Conceptualization; methodology; resources; supervision; writing—original draft; writing—review & editing.

## CONFLICT OF INTEREST STATEMENT

The authors declare no conflict of interest.

## ETHICS STATEMENT

Ethics approval and informed consent not sought. This manuscript is a systematic review protocol, neither approval from the ethics committee nor informed consent from the study populations is required.

## TRANSPARENCY STATEMENT

The lead author Patricia Egwumba affirms that this manuscript is an honest, accurate, and transparent account of the study being reported; that no important aspects of the study have been omitted; and that any discrepancies from the study as planned (and, if relevant, registered) have been explained.

## Data Availability

This is a protocol paper; therefore, data sharing is not applicable to this paper as no datasets were generated or analysed in this current paper.
